# Utilization of Telehealth Services in Libya in Response to the COVID-19 Pandemic: Cross-sectional Analysis

**DOI:** 10.2196/23335

**Published:** 2021-02-26

**Authors:** Muhammed Elhadi, Ahmed Msherghi, Ahmed Elhadi, Aimen Ashini, Ahmed Alsoufi, Fatimah Bin Alshiteewi, Amna Elmabrouk, Ali Alsuyihili, Alsafa Elgherwi, Fatimah Elkhafeefi, Sarah Abdulrazik, Ahmed Tarek

**Affiliations:** 1 Faculty of Medicine University of Tripoli Tripoli Libyan Arab Jamahiriya; 2 Faculty of Medicine University of Benghazi Benghazi Libyan Arab Jamahiriya

**Keywords:** COVID-19, cross-sectional study, resource-limited countries, SARS-CoV-2, telehealth, telemedicine, transitional countries, usability

## Abstract

**Background:**

Health care systems in transitional countries have witnessed unprecedented challenges related to adequate and continuous health care provision during the COVID-19 pandemic. In many countries, including Libya, institutions and organizations have begun to implement telehealth technology for the first time. This serves to establish an alternative modality for direct physician-patient interviews to reduce the risk of COVID-19 transmission.

**Objective:**

This study aimed to assess the usability of telehealth services in Libya and to provide an overview of the current COVID-19 scenario.

**Methods:**

In this cross-sectional study, an anonymous web-based survey was administered to Libyan residents between April and May 2020. Participants were contacted through text messaging, emails, and social media. The survey items yielded information on the sociodemographic characteristics, availability and accessibility of health care services, effects of the COVID-19 pandemic on health care services, mental health status, and the feasibility and application of the telehealth system.

**Results:**

We obtained 2512 valid responses, of which 1721 (68.5%) were from females. The participants were aged 28.2 (SD 7.6) years, of whom 2333 (92.9%) were aged <40 years, and 1463 (58.2%) were single. Regarding the health care services and their accessibility, 786 (31.1%) participants reported having a poor health status in general, and 492 (19.6%) reported having a confirmed diagnosis of at least one chronic disease. Furthermore, 498 (19.9%) participants reported varying degrees of difficulty in accessing health care centers, and 1558 (62.0%) could not access their medical records. Additionally, 1546 (61.6%) participants experienced problems in covering medical costs, and 1429 (56.9%) avoided seeking medical care owing to financial concerns. Regarding the feasibility of the telehealth system, approximately half of the participants reported that telehealth services were useful during the COVID-19 pandemic, and 1545 (61.5%) reported that the system was an effective means of communication and of obtaining health care services. Furthermore, 1435 (57.1%) participants felt comfortable using the telehealth system, and 1129 (44.9%) felt that they were able to express themselves effectively. Moreover, 1389 (55.3%) participants found the system easy to understand, and 1354 (53.9%) reported having excellent communication with physicians through the telehealth system. However, only 1018 (40.5%) participants reported that communication was better with the telehealth system than with traditional methods.

**Conclusions:**

Our study revealed high levels of usability and willingness to use the telemedicine system as an alternative modality to in-person consultations among the Libyan residents in this study. This system is advantageous because it helps overcome health care costs, increases access to prompt medical care and follow-up evaluation, and reduces the risk of COVID-19 transmission. However, internet connectivity and electricity issues could be a substantial barrier for many resource-limited communities, and further studies should address such obstacles.

## Introduction

Since December 2019, COVID-19 has disrupted health care systems in many countries [1,2]. SARS-CoV-2, the causative virus of the severe viral pneumonia COVID-19, has infected more than 14,000,000 individuals and resulted in approximately 600,000 deaths worldwide [3,4].

The emergence of the COVID-19 pandemic has posed unprecedented challenges on health care systems in many countries, resulting in the cancellation of surgical procedures, closure of clinics, and an increase in the burden on health care services from patients with COVID-19 who require careful consideration and more care [5-7]. In response to this disruption, many countries have started implementing strategies to reduce the risk of disease transmission and to provide adequate health care services, especially among individuals with chronic diseases who need more care [8-11].

Therefore, many countries have started using telehealth services; that is, advanced technologies such as video, audio, and other means to provide health care services and promote the well-being of individuals while being physically distant from the health care provider [12-14]. Telehealth services offer several advantages to both patients and health care providers. The benefits of this system among patients include a shorter waiting time, a reduced need to travel long distances from their homes for health consultations, total avoidance of transport, and a reduced risk of infection, especially during the COVID-19 pandemic. The telehealth system offers several advantages to health care workers, including a marked reduction in patient interaction, the ability to assess patients with different diseases, the ability to perform follow-up evaluation and record outcomes, and a reduction in stress related to the COVID-19 pandemic.

The Libyan health care system operates primarily through public health centers and primary care centers, at which health services are provided at no cost. However, this manner of health care provision lacks sophisticated and advanced health services. In addition, a large number of private centers provide paid health services that may confer more advantages over traditional public health care services. However, these expensive private care services might represent a barrier to care provision among many Libyan residents. Although some large companies provide health insurance to their employees, such as policies that cover the main services provided by private health care centers, this provision is limited to a very specific portion of the population.

The Libyan health care system was not prepared for the COVID-19 pandemic. Hospitals and health care workers in Libya need better training and more resources to combat the pandemic, and there have been shortages of personal protective equipment, a lack of health services, and few prepared intensive care units [15,16]. These difficulties, along with the current ongoing civil war, pose challenges on the Libyan health care system, resulting in a high demand for urgent and determined action [17].

Many institutions and organizations have initiated telehealth programs for the first time during the COVID-19 pandemic in Libya to help patients avail of health care services and consultations without the need to visit hospitals and to reduce the risk of COVID-19 transmission. Since this is the first time telehealth services have been introduced in Libya, their usability by the Libyan population and their health-related effects need to be assessed, and the challenges faced by health care authorities and organizations in implementing these new strategies need to be determined. In this study, we aimed to assess the usability of telehealth services in Libya and to provide an overview of the current COVID-19 scenario.

## Methods

### Participant Recruitment

In this cross-sectional study, we distributed an anonymous web-based survey among Libyan residents between April and May 2020 via text message, email, and social media platforms. The inclusion criteria were that participants should be Libyan residents aged over 18 years. Incomplete questionnaires with >30% missing data were excluded from the analysis. We marked essential questions with the “required” function to ensure high quality of the data collected.

### Measurement Tool

The web-based survey was conducted in accordance with the Checklist for Reporting Results of Internet E-Surveys [18]. The first section of the survey included questions on demographic characteristics such as age, gender, social status, economic stability, residential status, smoking status, and general health. The second section of the survey comprised questions on the availability and accessibility of health care services, including the distance travelled to access nearest health care facility and the time taken to reach it, mode of transport available, and ability to cover medical costs. The third section comprised questions related to the effects of the COVID-19 pandemic on health care services. A subsequent section contained questions on mental health status, screening symptoms of anxiety and depression using the self-administered 9-item Patient Health Questionnaire (PHQ-9) [19,20], wherein a score of ≥15 was considered the threshold for depression. The 7-item Generalized Anxiety Disorder scale [21] was used to assess anxiety, with a score of ≥15 as the threshold for anxiety. The final section of the questionnaire assessed the usability of telehealth in accordance with the Telehealth Usability Questionnaire devised by Parmanto et al [22] and validated by Zhou et al [23]. The telehealth questionnaire was translated to Arabic by two independent translators using the forward-backward translation method. Any discrepancy in the translated versions was resolved through further discussion until consensus was reached. The translated questionnaire was piloted with 30 participants, and the internal consistency was determined from a Cronbach *α* of .74 in the translated Arabic version. The telehealth usability survey included 3 items on the usefulness of telehealth, 3 items on the ease of use and learnability, 4 items on interface quality, 4 items on the quality of interaction, 3 items on reliability, and 4 items on satisfaction. Each of these items was scored on a 3-point Likert scale (0=agree, 1=neutral, 2=disagree).

### Statistical Analysis

Descriptive statistics are presented as frequency and percentage values. Continuous variables are presented as mean (SD) values. We performed a chi-square test to investigate associations between basic study characteristics and gender. Statistical analyses were performed using SPSS software (version 25.0, IBM Corp). A *P* value of ≤.05 was considered significant

### Ethical Considerations

The study was approved by the Bioethics Committee of the Biotechnology Research Centre (Tripoli, Libya; 109.3-2020). Participants provided written informed consent and their anonymity was maintained.

## Results

### Participant Characteristics

We obtained 2512 valid responses. In total, 1721 (68.5%) respondents were female and 791 (31.5%) were male. The study participants were aged 28.2 (SD 7.6) years. Most participants (n=2333, 92.9%) were aged <40 years and 1463 (58.2%) were single. In total, 766 (30.5%) participants were students and constituted the majority of the cohort by occupation. A total of 492 (19.6%) respondents reported having a chronic disease, 1137 (45.3%) reported having an excellent health status, and 786 (31.1%) reported having a poor health status. Table 1 summarizes the baseline demographic characteristics of the study population. We observed a significant association between gender and age (*P*<.001), marital status (*P*=.002), employment status (*P*<.001), stable income (*P*=.03), smoking status (*P*<.001), chronic disease (*P*=.02), and living arrangement (*P*=.03).

**Table 1:** Baseline demographic characteristics of the study population (N=2512).

Variables		Total, n (%)	Female (1721), n (%)	Male (n=791), n (%)	*P* value
**Age range (years)**					<.001^a^
	18-25	1062 (42.3)	748 (43.5)	314 (39.7)	
	26-40	1271 (50.6)	878 (51.0)	393 (49.7)	
	>40	179 (7.1)	95 (5.5)	84 (10.6)	
**Marital status**					.002^b^
	Married	971 (38.7)	707 (41.1)	264 (33.4)	
	Single	1463 (58.2)	960 (55.8)	503 (63.6)	
	Divorced	58 (2.3)	39 (2.3)	19 (2.4)	
	Widowed/widower	20 (0.8)	15 (0.9)	5 (0.6)	
**Employment status**					<.001^a^
	Student	766 (30.5)	527 (30.6)	239 (30.2)	
	Business and management	180 (7.2)	119 (6.9)	61 (7.7)	
	Engineering and manufacturing	68 (2.7)	42 (2.4)	26 (3.3)	
	Health care	315 (12.5)	213 (12.4)	102 (12.9)	
	Teaching and education	266 (10.6)	205 (11.9)	61 (7.7)	
	Science and pharmaceutical	65 (2.6)	47 (2.7)	18 (2.3)	
	Retails and sales	12 (0.5)	3 (0.2)	9 (1.1)	
	Homemaker	377 (15.0)	327 (19.0)	50 (6.3)	
	Laborer	24 (1.0)	17 (1.0)	7 (0.9)	
	Freelance	54 (2.1)	10 (0.6)	44 (5.6)	
	Retired	4 (0.2)	2 (0.1)	2 (0.3)	
	Unemployed	316 (12.6)	177 (10.3)	139 (17.6)	
	Other	65 (2.6)	32 (1.9)	33 (4.2)	
Stable income		846 (33.7)	556 (32.3)	290 (36.7)	.03^b^
Smoking		104 (4.1)	35 (2.0)	69 (8.7)	<.001^a^
Having chronic disease		492 (19.6)	329 (19.1)	163 (20.6)	.02^b^
**Living arrangement**					.03^b^
	With family	2065 (82.2)	1396 (81.1)	669 (84.6)	
	Alone	447 (17.8)	325 (18.9)	122 (15.4)	
**General health status**					.40
	Excellent	1137 (45.3)	766 (44.5)	371 (46.9)	
	Very good	61 (2.4)	44 (2.6)	17 (2.1)	
	Good	528 (21.0)	376 (21.8)	152 (19.2)	
	Bad	786 (31.1)	535 (31.1)	251 (31.7)	

^a^*P*<.001.

^b^*P*<.05.

### Accessibility of Health Care Services

Most participants reported an adequate level of ease in accessing health care centers, with 715 (28.5%) responding with “very easy” and 1299 (51.7%) responding with “easy.” Regarding their mode of transport, almost half of the participants (n=1140, 45.4%) reported driving their own car, and 1097 (43.7%) hired a taxi or a private driver. In addition, 989 (39.4%) respondents reported that it took them 15-30 minutes to reach a health care facility, whereas 804 (32%) reported that they needed less than 15 minutes to reach their nearest facility. Furthermore, 1407 (56%) participants reported that they could consult with a specialist physician within 2 days, whereas 1558 (62%) reported that they could not access their medical records or files.

Most participants (n=1546, 61.6%) found it difficult to cover the costs of medical care. More than half of the participants (n=1429, 56.9%) reported that they avoided seeking medical care for the fear of being financially burdened. Some participants (n=1225, 48.7%) reported difficulties in availing of emergency health care services. Most participants (n=2171, 86.4%) agreed that the working hours of clinics needed to be extended; however, approximately 1264 (50.4%) reported that it was easy to interact with physicians and nurses during health care counseling, and the remaining 1248 (49.6%) participants responded with “neutral” or disagreed with the statement, “Interactions with nurses and doctors are easy.” The findings of the assessment of the accessibility of health care services are summarized in Table 2.

**Table 2:** Accessibility of health care services among the study participants (N=2512).

Component		Total, n (%)
**Ease of traveling to the health care center**		
	Very easy	715 (28.5)
	Somewhat easy	1299 (51.7)
	Slightly difficult	441 (17.6)
	Very difficult	57 (2.3)
**Mode of transport to the health care center**		
	Private car	1140 (45.4)
	Friend’s or relative’s car	83 (3.3)
	Public transport	93 (3.7)
	Taxi or private driver	1097 (43.7)
	Walking	99 (3.9)
**How long does it take to reach the health care facility?**		
	<15 minutes	804 (32.0)
	15-30 minutes	989 (39.4)
	31-45 minutes	362 (14.4)
	46-60 minutes	129 (5.1)
	1-2 hours	132 (5.3)
	>2 hours	96 (3.8)
**If you get sick and need to see a specialist physician, how long does it take to get an appointment?**		
	≤2 days	1407 (56.0)
	3 days to 1 week	704 (28.0)
	1-2 weeks	228 (9.1)
	3-4 weeks	70 (2.8)
	>4 weeks	103 (4.1)
**The health care center provides me with the results of all my laboratory tests**		
	No	615 (24.5)
	Rarely	193 (7.7)
	Sometimes	536 (21.3)
	Usually	568 (22.6)
	Always	600 (23.9)
**If I want to see my medical records, the health care center allows me to have them**		
	No	1558 (62.0)
	Rarely	172 (6.8)
	Sometimes	276 (11.0)
	Usually	207 (8.2)
	Always	299 (11.9)
**I find it difficult to cover my medical care costs**		
	Strongly disagree	42 (1.7)
	Disagree	220 (8.8)
	Neutral	704 (28.0)
	Agree	899 (35.8)
	Strongly agree	647 (25.8)
**I avoid seeking medical care owing to financial concerns**		
	Strongly disagree	106 (4.2)
	Disagree	396 (15.8)
	Neutral	581 (23.1)
	Agree	746 (29.7)
	Strongly agree	683 (27.2)
**I find it challenging to obtain prompt medical counseling in emergencies**		
	Strongly disagree	83 (3.3)
	Disagree	392 (15.6)
	Neutral	812 (32.3)
	Agree	797 (31.7)
	Strongly agree	428 (17.0)
**Hospital care is always accessible without difficulty**		
	Strongly disagree	119 (4.7)
	Disagree	479 (19.1)
	Neutral	777 (30.9)
	Agree	849 (33.8)
	Strongly agree	288 (11.5)
**I can get medical advice whenever I need it**		
	Strongly disagree	70 (2.8)
	Disagree	465 (18.5)
	Neutral	842 (33.5)
	Agree	879 (35.0)
	Strongly agree	256 (10.2)
**Working hours of clinics should be further extended**		
	Strongly disagree	0 (0)
	Disagree	49 (2.0)
	Neutral	292 (11.6)
	Agree	1001 (39.8)
	Strongly agree	1170 (46.6)
**Interactions with nurses and physicians are easy**		
	Strongly disagree	130 (5.2)
	Disagree	516 (20.5)
	Neutral	602 (24.0)
	Agree	853 (34.0)
	Strongly agree	411 (16.4)

### Impact of the COVID-19 Pandemic on the Participants’ Health

Only 90 (3.6%) participants agreed that the lockdown had affected their health, while most participants (n=2207, 87.9%) disagreed with that statement. Most participants (n=1264, 86.8%) disagreed with the statement that the lockdown had psychological effects. However, 1531 (60.9%) participants were worried about being afflicted with COVID-19, and 1974 (78.6%) were worried about their family members becoming infected. Table 3 provides an overview of the effects of COVID-19 on the survey participants.

**Table 3:** Effects of the COVID-19 pandemic on the health of the study participants (N=2512).

Component		Total, n (%)
**The lockdown affected my health**		
	Strongly disagree	1241 (49.4)
	Disagree	966 (38.5)
	Neutral	215 (8.6)
	Agree	90 (3.6)
	Strongly agree	0 (0)
**The lockdown markedly affected my psychological status**		
	Strongly disagree	1155 (46.0)
	Disagree	1025 (40.8)
	Neutral	138 (5.5)
	Agree	194 (7.7)
	Strongly agree	0 (0)
**Are you worried about getting COVID-19?**		
	Yes	1531 (60.9)
	No	981 (39.1)
**Are you worried about the current government policies and responses of the society members to prevent and control the spread of COVID-19?**		
	Yes	2004 (79.8)
	No	508 (20.2)

### Mental Health Outcomes

In total, 454 (18.1%) participants had scores of ≥15 on the PHQ-9, which indicates that they had symptoms of depression. Furthermore, 382 (15.2%) participants had scores of 20-27 on the PHQ-9, which indicated a high likelihood among these participants to experience symptoms of depression. In addition, 372 (14.8%) participants had scores of ≥15 on the 7-item Generalized Anxiety Disorder scale, which indicates that they had anxiety symptoms.

### Telehealth Usability

Approximately half of the survey respondents agreed on the usefulness of telehealth services during the COVID-19 pandemic. In addition, more than half of the study participants reported that it was easy to avail of telehealth services and trusted this type of health care service. More than half of the study participants (n=1435, 57.1%) reported that they felt comfortable seeking the telehealth services, and 1389 (55.3%) reported that the system was easy to understand. Furthermore, 1354 (53.9%) participants reported that they could easily communicate with physicians through the telehealth system. However, only 1018 (40.5%) participants reported that communication with physicians was easier through the telehealth system than through other traditional methods. Regarding the reliability of the system, 1034 (41.2%) participants reported that the efficiency of the system was similar to that of in-person physician consultations, while 1175 (46.8%) participants responded with “neutral” to this statement. Additionally, 1439 (57.3%) participants felt comfortable communicating with physicians through the telehealth system, while 1545 (61.5%) reported that the system provides an effective means of communication when availing health care services. More than half of the participants (n=1383, 55.1%) felt satisfied with the telehealth system and were willing to use it in future. Table 4 provides an overview of the participants’ perception of the usability of telehealth services during the COVID-19 pandemic.

**Table 4:** Usability of telehealth services among the study participants (N=2512).

Component		Agree, n (%)	Neutral, n (%)	Disagree, n (%)
**Usefulness**				
	The telehealth system has improved my access to health care services	1134 (45.1)	1058 (42.1)	320 (12.7)
	The telehealth system saves me the time I spend going to a hospital, clinic, or specialist	1407 (56.0)	959 (38.2)	146 (5.8)
	The telehealth system satisfies all of my health care needs	1331 (53.0)	978 (38.9)	203 (8.1)
**Ease of use and learnability**				
	The system is simple and easy to use	1479 (58.9)	877 (34.9)	156 (6.2)
	It was easy to learn how to use the system	1348 (53.7)	1024 (40.8)	140 (5.6)
	I trust that I can quickly get results with this system	1367 (54.4)	979 (39.0)	166 (6.6)
**Interface quality**				
	Handling this system is very comfortable to me	1435 (57.1)	959 (38.2)	118 (4.7)
	I like to use this system	1224 (48.7)	1073 (42.7)	215 (8.6)
	The system is simple and easy to understand	1389 (55.3)	974 (38.8)	149 (5.9)
	The system is able to perform all the tasks that I want it to perform	1158 (46.1)	1142 (45.5)	212 (8.4)
**Interaction quality**				
	I can easily talk to a physician when using the telehealth system	1354 (53.9)	942 (37.5)	216 (8.6)
	I can clearly hear the doctor when using the telehealth system	1048 (41.7)	1194 (47.5)	270 (10.7)
	I felt able to express myself effectively	1129 (44.9)	898 (35.7)	485 (19.3)
	Seeing a physician through the telehealth system was as easy as having an in-person consultation	1018 (40.5)	1247 (49.6)	247 (9.8)
**Reliability**				
	Physician consultations using the telehealth system were as efficient as in-person consultations	1034 (41.2)	1175 (46.8)	303 (12.1)
	If any error occurs when using the system, I can correct the error quickly and easily	1225 (48.8)	1072 (42.7)	215 (8.6)
	The system sends messages when errors occur, and that precisely guided me on how to correct errors	1444 (57.5)	902 (35.9)	166 (6.6)
**Satisfaction and future use**				
	I felt comfortable when communicating with a physician through the telehealth system	1439 (57.3)	855 (35.2)	188 (7.5)
	The telehealth system is an acceptable way to receive health care services	1545 (61.5)	785 (31.3)	182 (7.2)
	I will use the telehealth service again in the future	1383 (55.1)	949 (37.8)	180 (7.2)
	In general, I am completely satisfied with the telehealth system	1451 (57.8)	861 (34.3)	200 (8.0)

## Discussion

### Principal Findings

This study aimed to assess the accessibility, applicability, and feasibility of telehealth services in Libya during the COVID-19 pandemic. Most study participants reported being able to access health care facilities. However, most participants found it difficult to cover the cost of the health care services, and more than half of them avoided seeking these services owing to the difficulty in covering their costs. Our study participants reported high levels of telehealth usability and applicability. More than half of the participants (n=1435, 57.1%) reported feeling comfortable using telehealth services, 1389 (55.3%) reported that the system was easy to use, and 1354 (53.9%) reported an enhanced level of interaction with health care providers. Furthermore, 1018 (40.5%) participants reported that communication with physicians was better through the telehealth system than through traditional health care methods, while 1034 (41.2%) reported that the telehealth services were equally as efficient as in-person health care services. In addition, our participants experienced moderate levels of symptoms of anxiety (n=372, 14.8%) and depression (n=382, 15.2%).

Regarding the accessibility of the telehealth services, most participants did not report any difficulty in in reaching health care facilities or arranging an appointment with a specialist. However, the participants reported difficulties in accessing their laboratory findings and hospital records, which may be influenced by the unavailability of electronic health records in most hospitals and the unavailability of laboratory facilities in several major hospitals, where patients have to visit another facility to obtain the results of laboratory tests.

### Comparison With Previous Studies

Our results suggest that the telehealth system potentially provides an alternative to traditional health care services, especially during the COVID-19 pandemic, concurrent with Mclean et al [24] who reported no difference in outcomes between traditional and telehealth services. Telehealth services offer more flexibility in securing appointments and are more comfortable for individuals living far from health care facilities, especially cancer patients or individuals with chronic diseases who might be at increased risk of severe COVID-19 [25,26]. However, further development of telehealth services is needed to supply the demand for several specialties that require a specific eHealth-based health care infrastructure [27]. A recent study from China [28] reported that telemedicine and telehealth services were effective and feasible and allowed for substantial improvement in health care services for patients with COVID-19. Another study from China [29] on the role of internet hospitals in the COVID-19 pandemic reported that telehealth services and internet hospitals can offer essential support in controlling the pandemic by reducing the cross-infection risk, thus curbing the spread of COVID-19. However, several barriers and challenges regarding the implementation of internet hospitals remain, and further studies with large samples are required to focus on testing specific prototype internet hospitals [30].

Another example of the efficiency of telehealth services is based on individuals with diabetes; telemedicine has been reported to substantially improve health-related outcomes among individuals with diabetes [31]. Another study that used big data and artificial intelligence approaches for diabetes care supported this finding [32].

Our study provides an overview of the mental health status of the Libyan population. Mental health services constitute a major application of telehealth, with several smartphone apps yielding positive outcomes during self-management of depression [33]. The demand for these interventions is high, given the current state of mental health during the COVID-19 pandemic, and telehealth facilitates follow-up evaluation of patients with psychiatric disorders without the need for hospital visits. On-demand mental health telehealth systems have shown promising outcomes in reducing the symptoms of depression [34].

Several challenges are associated with the implementation of telehealth services in Libya. The first issue relates to finances. The government and health care authorities need to provide support for telehealth services and provide funding opportunities to help new organizations and health care centers change their traditional approaches for care provision to a remote system to reduce the risk of COVID-19. This is critical because Libya is faced with a financial crisis, and it would be difficult for Libyan residents to cover the cost of such expensive health care services. Second, Libyan residents have been facing problems with electricity provision and internet access over the last few years. It would be majorly challenging to implement these services during the civil war when internet connectivity is troublesome.

Another issue that needs to be considered in Libya is the protection of patient confidentiality and the ability to devise methods that protect the personal and clinical data of patients. Our study clearly highlights this issue; 1558 (62.0%) participants reported that they could not access their health reports. However, it is also concerning that several private telehealth services are providing care without any formal agreements or specific policies to protect patient data or let patients access their own health records. Therefore, further studies should address this issue and focus on data protection and on providing patients easy access to their health data.

### Strengths and Limitations

This study has several strengths. First, to our knowledge, this study is the first to validate the Telehealth Usability Questionnaire as an instrument in Arabic. Second, this study is the first to provide an overview of the usability of telehealth and the health status of the Libyan population. Third, our study included a large sample of 2512 individuals with complete data and assessed several health-related issues with a special emphasis on accessibility and the mental health status of the Libyan population. Furthermore, to our knowledge, this study is the first to assess the usability of telehealth services during the COVID-19 pandemic in a transitional, resource-limited country. Our study furthers the current understanding of the usability of telemedicine in transitional, resource-limited countries, and our results support the implementation of telehealth programs despite certain potential challenges associated with them.

The study has several limitations that should be addressed in future studies. First, it was a cross-sectional survey carried out during the COVID-19 pandemic simultaneously with the introduction of telehealth services, and some participants may not have used telehealth services during this period. Second, we did not assess internet connectivity and electricity provision, which are potential factors affecting the ability of some communities to access telehealth services; these factors need to be addressed in future studies. Third, this study was an online cross-sectional survey, and most participants were young and without major comorbidities or chronic diseases; therefore, we may have overestimated the usability of these services because older individuals may face more challenges in using these technologies and may require more assistance. Therefore, further studies are required to assess elderly individuals and those with special needs and to investigate how telehealth systems could be implemented for these specific groups of patients. Fourth, we used self-reported scales, which may have increased the risk of bias. We also used a 3-point Likert scale for the Telehealth Usability Questionnaire; this may have influenced our findings.

### Conclusions

Telehealth services can substantially replace in-person consultations and prevent nosocomial infections in Libya during the COVID-19 pandemic. Our study provides an overview of the accessibility and usability of telehealth services, and our results suggest that telehealth services help reduce the workload of physicians and direct contact with patients during the COVID-19 pandemic. Our results support the implementation of telehealth services; however, further studies are required to focus on the protection of patient confidentiality and personal data.

## Abbreviations

PHQ-9: 9-item Patient Health Questionnaire
